# Blood serum retinol levels in Asinara white donkeys reflect albinism‐induced metabolic adaptation to photoperiod at Mediterranean latitudes

**DOI:** 10.1002/ece3.2613

**Published:** 2016-12-20

**Authors:** Maria Grazia Cappai, Maria Grazia Antonietta Lunesu, Francesca Accioni, Massimo Liscia, Mauro Pusceddu, Lucia Burrai, Maria Nieddu, Corrado Dimauro, Gianpiero Boatto, Walter Pinna

**Affiliations:** ^1^ Department of Agricultural SciencesUniversity of SassariSassariItaly; ^2^ Department of Chemistry and PharmacyUniversity of SassariSassariItaly; ^3^ Colonia Penale Is ArenasArbusItaly

**Keywords:** melanin, skin damage, sun radiation, vitamin A, β‐carotene

## Abstract

Previous works on albinism form of Asinara white donkeys (*Equus asinus*) identified the mutation leading to the peculiar phenotype spread to all specimens of the breed. Inbreeding naturally occurred under geographic isolation, on Asinara Island, in the Mediterranean Sea. Albino individuals can be more susceptible to develop health problems when exposed to natural sun radiation. Alternative metabolic pathways involved in photoprotection were explored in this trial. Nutrition‐related metabolites are believed to contribute to the conservation of Asinara donkeys, in which melanin, guaranteeing photoprotection, is lacking. Biochemical profiles with particular focus on blood serum β‐carotene and retinol levels were monitored. Identical natural grazing conditions for both Asinara (albino) and Sardo (pigmented) donkey breeds were assured on same natural pastures throughout the experimental period. A comparative metabolic screening, with emphasis on circulating retinol and nutrient‐related metabolites between the two breeds, was carried out over one year. Potential intra‐ and interspecimen fluctuations of metabolites involved in photoprotection were monitored, both during negative and positive photoperiods. Differences (*p *=* *.064) between blood serum concentrations of retinol from Asinara versus Sardo breed donkeys (0.630 *vs*. 0.490 μg/ml, respectively) were found. Retinol levels of blood serum turned out to be similar in the two groups (0.523 *vs*. 0.493 μg/ml, respectively, *p *=* *.051) during the negative photoperiod, but markedly differed during the positive one (0.738 vs. 0.486, respectively, *p *=* *.016). Blood serum β‐carotene levels displayed to be constantly around the limit of sensitivity in all animals of both breeds. Variations in blood serum concentrations of retinol in Asinara white donkeys can reflect the need to cope with seasonal exposure to daylight at Mediterranean latitudes, as an alternative to the lack of melanin. These results may suggest that a pulsed mobilization of retinol from body stores occurs to increase circulating levels during positive photoperiod.

## 1 Introduction

**Figure 1:**
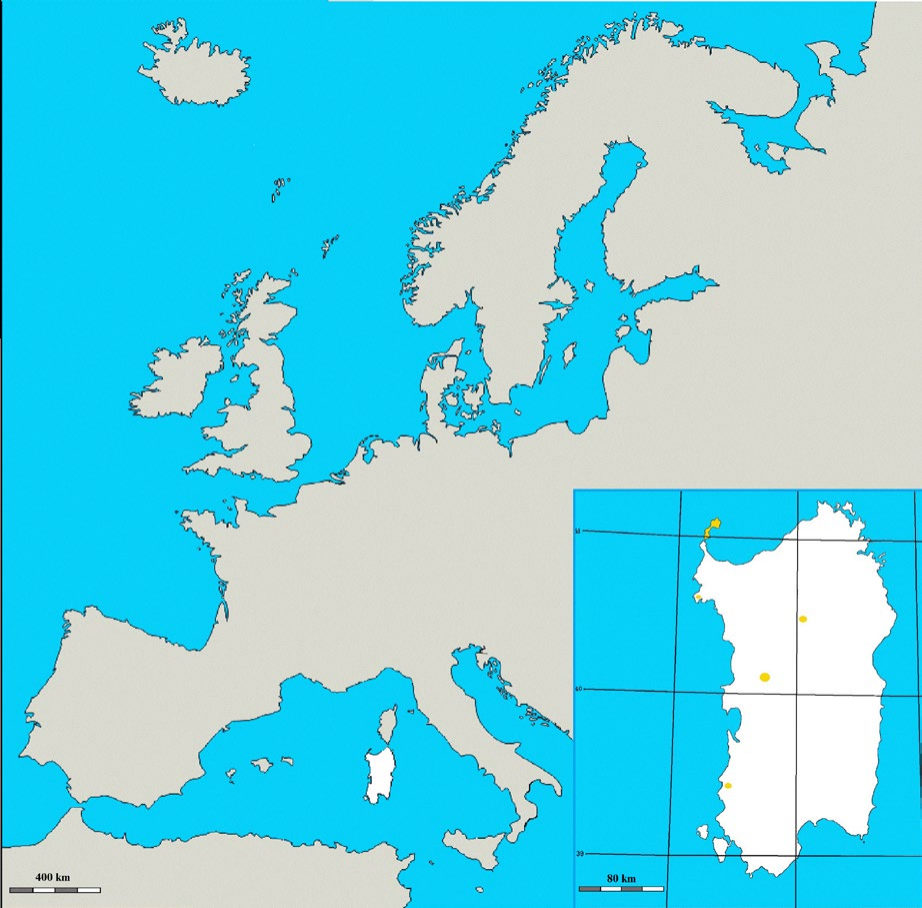
Map of Europe and magnification of Sardinia Isle (white), one of the largest islands of the Mediterranean Sea. In the frame on the right, the Asinara Island is colored in yellow and yellow spots on Sardinia highlight the presence of Asinara white donkeys in other regional parks. The National Registry of Local Minor Equine Breeds accounts 94 Asinara white donkeys living in Sardinia in 2015

**Figure 2:**
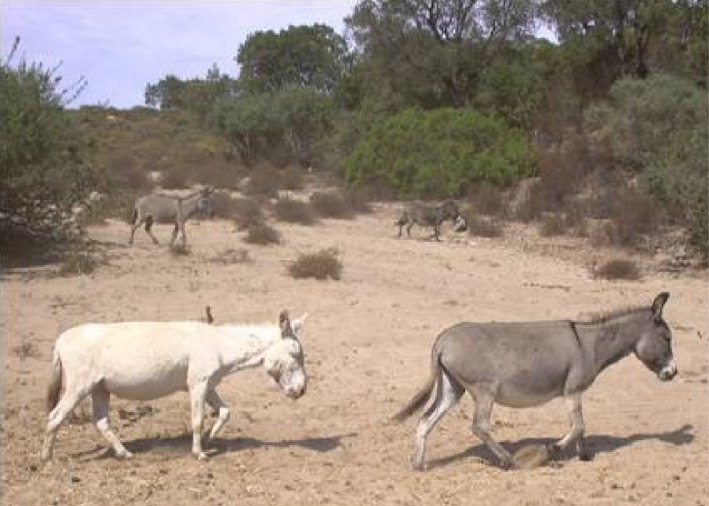
Group of donkeys in the natural park in Is Arenas. Asinara white donkey (bottom left) and Sardo donkeys (bottom right and in the background) stepping toward bushes of Mediterranean vegetation. Picture taken during positive photoperiod

A worldwide acknowledged unique breed of feral albino donkeys originated in Sardinia, one of the major islands of the Mediterranean Sea. The Asinara white donkeys (*Equus asinus*, Linnaeus, 1758, var. albino) owe their name to the Asinara Island (N 41°4′ 0.012″, E 8°16′ 0.012″, Sardinia, Italy), established as National Park of the Autonomous Region of Sardinia, since 1998 (Official Gazette of Italian Republic, 1997) (Figure 1). The peculiar phenotype of Asinara white donkeys is characterized by a lifelong hypopigmentation of skin, hair, and eyes (Figure 2). The coat color has been definitely assessed (Cappai et al., 2015), and the relative mutation has been recently elucidated (Utzeri et al., 2015). All specimens of Asinara breed display to possess an oculocutaneous form of albinism, classified as type 1 (OCA1). In OCA1 albinism, the phenotype can be due to the impaired conversion of L‐tyrosine to L‐3,4‐dioxyphenylalanine (L‐DOPA), in the early steps of the melanogenic process. The enzymatic inactivity (Cappai et al., 2015) of tyrosinase (TYR) is now known to be due to the genetic mutation (Utzeri et al., 2015) of tyrosinase gene (*Tyr*).

The consistency of Asinara white donkeys living on the Asinara Island is currently estimated to account 140 individuals. A lesser number of donkeys is distributed in other national parks of the Autonomous Region of Sardinia territory and, residually, in the rest of Italy (specimens: 294; conservation status: critical; records of the National Registry of Local Minor Equine Breeds, Association of Italian Breeders, 2016).

Previous phylogenetic analyses (Cosseddu et al., 2001; Pinna, Cosseddu, Moniello, & Zimdars, 1998) suggested that the fixation of the mutation for albinism has been favored by geographical isolation. The autochthon origin of Asinara breed was supported by the molecular analyses carried out by Pinna et al. (1998) who reported that albino donkeys seem to have branched out of the autochthon pigmented Sardo donkey, later studied also by Cosseddu et al. (2001). The first description of the Asinara white donkeys dates back to 19th century. This discovery can support the theory about the role exerted by geographic isolation and inbreeding phenomena. Inbreeding can naturally take place when genetically related individuals mate and offspring carry high levels of consanguinity. This may result in homozygosis for recessive alleles which are fixed in the next generations. The albinism of Asinara white donkeys has been recently identified to be due to a missense mutation in a highly conserved aminoacid position (G/G or D/D genotype), diverse from the pigmented phenotype (grey) of the Sardo donkey (C/C or C/G genotype) (Utzeri et al., 2015). Inbreeding was also associated with isolated forms of albinism in other animal species (Prado‐Martinez et al., 2013; Protas et al., 2006).

In individuals with different types of albino forms, the risk to develop skin cancer is reported to be higher than observed in pigmented individuals. In addition, this datum varies according to the type of albinism (Grønskov, Ek, & Brondum‐Nielsen, 2007; Okulicz, Shah, Schwartz, & Janninger, 2003; Prado‐Martinez et al., 2013). Other disorders are referred to ophtalmological problems like nystagmus or epiphora following direct sun exposure. Asinara donkeys show pink or light blue iris and unpigmented ocular *fundus* (Cappai et al., 2015; Pinna et al., 1998). As melanin is a skin, eye (retina and iris), and hair pigment with photoprotection properties against UV radiation, albino individuals may be naturally prone to photosensitivity and related complications. A high prevalence of secondary skin or eye disorders is reported to be linked to the form of albinism (Oetting, 2000; Oetting, Brilliant, & King, 1996; Potterf et al., 1998; Witkop, 1971). Despite Asinara donkeys display OCA1 phenotype (Cappai et al., 2015), the prevalence of skin or eye diseases is not reported to be higher than that detectable in other pigmented feral donkeys living in the same environment, except for photodermatitis at the top of the ears (Cappai et al., 2015) and lower resistance to *Myiasis cutanea* (Pinna, Vacca, Cubeddu, Pintori, & Garippa, 1994).

Against this background, Asinara white donkeys’ adaptation to the natural Mediterranean environment poses the question on how OCA1 does not seem to induce secondary diseases incompatible with life and responsible for low survival rates in nature, like skin cancer for instance, never reported in the literature to the best our knowledge. It appeared highly stimulating to understand how these albino animals successfully adapted to the Mediterranean climate and coped with environment and natural feeding stuffs. It was therefore hypothesized that endogenous factors, other than melanin, may play a decisive role in the natural metabolic adaptation of Asinara white donkeys. In the present work, we investigated whether variations in circulating retinol, as a biological measure to overcome the lack of pigment in skin, eye, and hair of Asinara white donkeys could be detected. Carotenoids consumed with the natural diet seem to be linked to circulating levels and distribution of metabolically active derivates in tissues of fish, birds, and mammals, and retinol may be one of these. In general, carotenoids may serve different purposes in the animal body, with different evolutionary meanings (McGraw, Nolan, & Crino, 2011). Dietary carotenoids display to possess diverse chemical structures. To date, over 600 known carotenoids compose this heterogeneous group of pigments, of which about 50 are useful substances for plants and animals. Carotenoids can be synthesized by plants, algae, and fungi, but mammals are incapable of *de novo* synthesis (Hammond & Renzi, 2013). The diverse chemistry can be used to explain how different carotenoids enter diverse biochemical pathways. As a consequence, different physiological activities can lead to diverse biological effects of carotenoids after ingestion (Hammond & Renzi, 2013). Due to their widespread presence in nature, carotenoids were the first phytochemicals studied for their ubiquitous functional roles (Eugster, 1995). Carotenoids can be classified into carotenes (hydrocarbons, likewise β‐carotene and lycopene) and their oxidation products, known as xanthophylls. With regard to xanthophylls, they are known to contribute to the plumage of birds and coloration of fish. In particular, seasonal variations (nonmolt and molt, for instance) were observed in finches differently supplemented with dietary carotenoids (McGraw et al., 2011). To such an extent, the involvement of carotenoids in different aspects of life appears to be a common trait both for animals and plants. Vegetables and fruits are major sources of carotenoids for terrestrial mammals; however, other carotenoids (astaxanthin, for istance) can be synthesized by krill (Katsuyama, Komori, & Matsuno, 1987), thus being naturally available in the diet of marine animals and aquatic birds, like flamingos (Fox, 1955). Natural pigments in the plant kingdom are associated with photosynthetic processes, as well as to dissipate energy excesses under light stress. The latter condition is mediated by a particular group of carotenoids (Delgado‐Vargas, Jiménez, & Paredes‐Lòpez, 2000; Demmig, Winter, Krüger, & Czygan, 1987; Demmig‐Adams & Adams, 1996). Carotenes from plants, in particular β‐carotene, display to possess pro‐vitaminic properties for mammals. Vitamin A, or retinol, is synthesized starting from β‐carotene in the small intestine and liver of animals. Vitamin A is known to take part, among other biological activities, in the visual function following light exposure. Vitamin A behaves as a strong antioxidant, capable to preserve cell membrane integrity. The role of retinol and β‐carotene was explored by Wolf et al. (2000) in white recessive canaries, but no albino forms were associated in these birds. Thus, a comparative trial between Asinara white and pigmented Sardo donkeys, kept under same natural conditions and equally exposed to different intensities and duration of natural daylight, was carried out, with the attempt to clarify whether retinol might take part in photoprotection of albino specimens. Thus, this investigation was carried out over one year, to explore the metabolic adaptation to Mediterranean climate of albino specimens during different photoperiods.

## 2 Materials and methods

### 2.1 Animals and care

The investigation involved 11 stallions and 12 jennies, of which six of Asinara white and 17 of Sardo breed. All animals were individually recorded in the Official Register of the white donkey of Asinara (Ministerial Decree 27/7/1990) and of Sardo breed, respectively. The proportion of specimens from each breed enrolled in the trial is representative of the consistency of Asinara and Sardo donkeys, namely hundreds and thousands, respectively.

All the experimental procedures presented in this study comply with recommendations of European Union directive 86/609/EEC and Italian law 116/92 concerning animal care.

### 2.2 Animals and farming conditions

All donkeys enrolled in the trial live altogether in the natural reserve of Is Arenas, in the south‐western coast of Sardinia Island (N 41°4′0.012″, E 8°16′0.012″). Individual blood sampling was carried for determining overall metabolic profile, with particular regard to β‐carotene and retinol concentration, in two different periods (negative *vs*. positive photoperiod) throughout the year. Sampling periods were characterized by positive (peak in June, after three months of increasing light hours per day) and negative (month of October, after three months of decreasing light hours per day) photoperiod of the boreal hemisphere. All animals were individually and electronically identified by injectable transponders according to the EC Regulation 504/2008. Each donkey had free access to same natural pastures in both seasons.

At each sampling, donkeys underwent the nutritional assessment (Cappai, Picciau, & Pinna, 2013) to estimate the nutritional state. Moreover, the comparative approach for the nutritional assessment was used to estimate any potential nutritional deficiency clinically manifest in both breeds. The trial lasted 12 months, which was considered a reliable period of time for avoiding biases due to bioaccumulation in the body of fat soluble dietary compounds from earlier seasons.

### 2.3 Blood sampling and laboratory analysis

Each donkey from both breeds was sampled for whole blood through the puncture of the jugular vein, to screen complete biochemical profile at start (October 2013) and end of the trial (October 2014), during negative photoperiod. Blood samples were also collected from same animals in the month of June 2014 (positive photoperiod), between the two negative photoperiods. For this purpose, all animals were gathered together in a paddock with mobile fences and moved into a corridor leading to a horse stock.

Individual blood samples were cooled down and stored in tubes of polystyrene cases in the upright position in a cooling bag, to assure adequate temperature during the transfer of samples to the laboratory. All samples were labeled with the donkey name, electronic individual code (EIC), and date of sampling. All laboratory procedures on whole blood were started within six hours after collection. In field and laboratory protocols for the collection, storage and analyses of blood samples were carried out in the dark, in order to avoid photo‐degradation of β‐carotene and retinol.

Individual serum was screened for complete biochemical profile. Prior to chemical analysis of blood serum, individual blood samples were centrifuged at 1500 × g for 10 min. An aliquot of individual serum was stored in a sterile vial (2 ml) and frozen at −20°C, until further analyses. All the samples were analyzed within one week, through an automatic biochemical analyzer (Mindray BS‐200, Alcyon, Italy) for the determination of serum concentration of ubiquitous intermediate metabolites, enzymes, nutrients, macro‐ and micro‐minerals.

For the determination of β‐carotene and retinol, high‐pressure liquid chromatography coupled with an ultraviolet detector (HPLC‐UV) was carried out. All standards and solvents were purchased from Sigma Aldrich (Milan, Italy). Stock solution (1 mg/ml) of β‐carotene and retinol were prepared in methanol and chloroform/methanol (50/50), respectively. For the calibration curve, standard stock solutions were diluted with methanol and kept frozen at −20°C, protected from light. Serum levels of β‐carotene and retinol were simultaneously measured at 325 and 450 nm, respectively.

Chromatographic separation was carried out on a Waters Symmetry C18 column (4.6 × 150 mm, particle size 5 μm, Waters, Milford, Massachusetts). The injection volume was 20 μl.

The mobile phases used were acetonitrile/methanol/Milli‐Q water (64.5/33/2.5) at 1 ml/min for retinol, and 100% methanol at 2.8 ml/min for β‐carotene.

Data were acquired and processed by Breeze Software (Waters, Milford, Massachusetts).

The limits of sensitivity for β‐carotene and retinol were 50 and 100 ng/ml, respectively.

Samples were prepared as follows: 0.3 ml of serum was vortexed with 0.6 ml of acetonitrile and centrifuged at 3500 × g at 4°C for 10 min. The supernatant was dried under a stream of nitrogen, and the residue was reconstituted in 0.15 ml of mobile phase (Biesalski, Greiff, Brodda, Hafner, & Bässler, 1986; Ganière‐Monteil et al., 1994; Gershkovich, Ibrahim, Sivak, Darlington, & Wasan, 2014; Levent, Oto, Ekin, & Berber, 2013; Milne & Botnen, 1986).

### 2.4 Analysis of data and statistical methods

Data obtained on each sampling were analyzed using the following linear model:

$$Y_{i,j}=\mu +D_{i}+G_{j}+D_{i}\times G_{j}+e_{i,j}$$

where *Y* is the dependent variable (β‐carotene and retinol concentration in blood serum), μ is the overall mean, *D* is the fixed effect of the sampling time (two levels: negative and positive photoperiod), *G* is the fixed effect of the coat color (two levels: pigmented *vs*. albino), D*G is the interaction factor, and *e* is the random residual. All data were analyzed using SAS 9.2 (SAS Inst. Inc. Cary, NC). The statistic significance was set for *p*‐value <.05. The *p*‐value <.10 represented a trend.

Correlation coefficients (r) and respective statistic *t*‐test (*p *<* *.05) were calculated for circulating Zn and total protein with retinol concentrations in blood serum. In both breeds, correlations were statistically analyzed because Zn and total protein were considered as nutrients related to intestinal absorption and conversion yields of β‐carotene.

## 3 Results

All animals involved in the trial appeared healthy. No signs of nutritional deficiency could be pointed out in both breeds. This finding was also supported by the optimal body condition score (BCS, based on a five‐point scale, 1 = emaciation to 5 = obesity) recorded both in Asinara and Sardo donkeys (3.25 ± 0.15 *vs*. 3.50 ± 0.10, respectively).

Biochemical profiles did not highlight significant differences between breeds (Table 1), as to parameters screened in this trial, except for retinol (Table 2). In fact, levels of retinol turned out to be significantly higher (+40.6% on average) in Asinara donkeys than those detected in blood serum of Sardo ones, during the positive photoperiod, whereas retinol concentrations (μg/ml) in blood serum appeared similar during negative photoperiods, in both the groups. The interaction between coat colour and photoperiod resulted to the limit of statistic significance (*p *=* *.051), as retinol blood serum concentrations in Asinara white donkeys set back to levels similar to those determined in Sardo donkeys during negative photoperiod. β‐Carotene levels constantly resulted around the limit of sensitivity in all animals (50 ng/ml). Correlations between retinol and circulating zinc and respective total protein were not statistically significant in both breeds (Table 3). This datum supports the consideration of a normal intestinal absorption of β‐carotene in both breeds.

**Table 1:** Biochemical metabolic profiles of donkeys (Asinara vs. Sardo) in blood serum collected during negative and positive photoperiods. Analyzed parameters involved in the nutritional assessment and potential impact on retinol metabolism are reported. Analyzed metabolites drop in the physiological range in both breeds

*Breed*	Asinara		Sardo	
*Coat*	Albino		Pigmented (gray)	
*Photoperiod*	Positive	Negative	Positive	Negative
*Animals*	6	17	6	17
*Parameter*				
Glucose (mg/dl)	38.8 ± 16.1	68 ± 7.91	53.0 ± 10.7	60.3 ± 4.84
Total protein (g/L)	82.3 ± 21.7	69.7 ± 2.61	84.0 ± 10.6	58.2 ± 7.27
Zinc (mg/dl)	40.6 ± 2.34	34.5 ± 2.12	44.1 ± 8.88	38.2 ± 2.19
Triglycerides (mg/dl)	67 ± 22.1	59.5 ± 17. 7	69.3 ± 23.2	60.4 ± 17.7
Cholesterol (mg/dl)	78.8 ± 20.3	75 ± 2.82	83.6 ± 5.65	87.2 ± 7.59
Urea (mg/dl)	42.0 ± 13.4	38.1 ± 1.27	48.8 ± 11.5	31.2 ± 9.54
Lipase (U/L)	16.1 ± 1.31	14.5 ± 0.22	16.2 ± 4.12	18.0 ± 1.87

**Table 2:** Retinol levels (μg/ml) in blood serum of Asinara versus Sardo donkeys during different photoperiods (negative vs. positive). Values are expressed as mean and pooled standard error (SE). Retinol concentrations in serum clearly indicate the increase of circulating retinol in the bloodstream of Asinara white donkeys during the positive photoperiod, if compared with retinol concentrations in serum from same animals during the negative photoperiod. By contrast, Sardo donkeys do not show any variations of circulating retinol levels across different photoperiods. Negative photoperiod concentrations of retinol from Asinara white donkeys are slightly higher than those determined in blood serum of Sardo donkeys

*Photoperiod*		Positive	Negative	
	Animals (*n*)	23	23	
*Breed*				SE
Asinara	6	0.738^a^	0.522^ab^	0.04
Sardo	17	0.486^b^	0.492^b^	0.03

Values that do not share a letter are significantly different (*p *<* *.05).

**Table 3:** Correlation coefficients and *p*‐values between circulating retinol, zinc (Zn), and total protein (TP) in Asinara white donkeys. No statistic significance was pointed out with blood serum concentrations of Zn and TP, whereas a statistically significant positive correlation was found between Zn and TP circulating levels

Correlations *p*‐value	Retinol (μg/ml)	Zn (mg/dl)
Zn (mg/dl)	−0.276	
	*0.172*	
TP (g/L)	−0.294	0.670
	*0.137*	*0.000*

## 4 Discussion

Herbivores fed on natural diets cannot consume adequate amount of retinol, necessary to cover their nutritional requirement, but they have to operate a conversion from its pro‐vitaminic form (β‐carotene) in the diet, from vegetal sources. As previously said, about 600 different compounds can be accounted in nature, of which only 50 can be found in human and animal diets. In particular, carotenes are hydrocarbons, thus their chains are only composed by C and H. Carotenoids containing an unsubstituted β‐ring and a C11 polyene chain are termed provitamin A, and they can display biological activities, once enzymatically converted in the animal body. Provitamin A carotenoids from plants are important sources of dietary vitamin A, or retinol, for herbivores; they can be found primarily in fresh vegetables and in some particular fruits. Bioaccessibility of β‐carotene from fat digestion, and its bioavailability, following the conversion into retinol, is genetically ruled (enzyme‐dependence) (Haskell, 2012; Jalal, Nesheim, Zulkarnain, Sanjur, & Habicht, 1998; Tang, 2012). Moreover, nutritional deficiencies of iron, zinc, and protein may also affect conversion rates of β‐carotene into vitamin A. Therefore, the bioavailability of retinol depends on the genetic type of individuals and on overall nutritional‐metabolic status. From a strict nutritional viewpoint, this is translated into a variable capability to digest, adsorb, and convert β‐carotene into retinol. The retinol can be later acquired by organs and tissues and stored in liver as retinyl esters (stellate cells mainly), retina (in the conversion of retinol–retinal–rhodopsin and reverse, for the visual function), fat and skin (Vahlquist, Lee, Michaëlsson, & Rollman, 1982). Previous studies in epithelial cells from skin of rats have shown that dietary retinoic acid supplementation induces transglutaminase activity, being this enzyme involved in programed cell death, and maybe involved in the inhibition of carcinogenesis (Jones et al., 1994). Intestinal conversion of β‐carotene to vitamin A decreases when an experimental oral dose of β‐carotene increases (Novotny, Harrison, Pawlosky, Flanagan, & Harrison,^2010^). This can be considered as a safe biological way to protect against the risk of the fat soluble vitamin A excesses, accumulated in tissues and organs, like the liver. In fact, it was seen that despite high intakes of β‐carotene, retinol levels do not increase proportionally (Novotny et al., 2010; Tourniaire et al., 2009). However, β‐carotene can be converted to retinol with different efficiency rates in the diverse animal species (Tourniaire et al., 2009). To the best of our knowledge, the conversion efficiency of β‐carotene into retinol was not experimentally determined in the donkey, but our results suggest that this species might be an efficient converter. It is known that different cleavage sites of β‐carotene molecule may give rise to diverse biochemical pathways, depending on symmetric cleavage by β‐carotene‐monooxygenase (β,β‐carotene‐15,15′‐monoxygenase 1, BCMO1), or eccentric cleavage operated by β‐carotene‐dioxygenase (β,β‐carotene‐9′,10′‐dioxygenase, BCDO2) (Lobo, Isken, Hoff, Babino, & von Lintig, 2012). Symmetric or eccentric cleavages give rise to a series of products from β‐carotene molecule, with diverse biological activities (Tourniaire et al., 2009). Thus, it is established that β‐carotene molecule does not produce retinol only (Tourniaire et al., 2009). Results obtained from this trial seem to suggest that both Asinara white and Sardo donkeys are efficient converters of β‐carotene into retinol. It could be argued that Asinara white donkeys may intake higher amounts of β‐carotene with the diet, by a more accurate selection of naturally available plant species. However, this aspect does not appear to be plausible given the nutritional status of animals from both breeds, which appeared similar throughout the experimental period. The intake of β‐carotene implies the consumption of proportional dietary fat with the diet, that would have led to different energy intake and consequent energy storage. Additionally, body condition scores together with variations associated with circulating total triglycerides in the bloodstream would have varied accordingly, but this was not found to differ between the two breeds. It was therefore considered that the metabolic response in the albino donkey can be elicited by increasing natural daylight exposure, namely during positive photoperiod. In fact, circulating retinol levels resulted higher in blood serum of Asinara white donkeys when compared to those determined in Sardo ones, during increasing intensity and duration of exposure to natural light. Such finding can be related to the key biological functions of vitamin A (retinol) and its aldehyde (retinaldehyde) in the visual function. The whole biochemical process leading to the involvement of retinol in the formation of rhodopsin is not reported here, as extensively reviewed by Palczewski (2006). Despite a comparable allowance to dietary β‐carotene from natural feeding sources available in the environment, Asinara white donkeys could efficiently mobilize retinol from tissue stores producing high circulating retinol levels in the bloodstream. This datum is supported by the circulating levels of retinol in Asinara donkeys during the negative photoperiod, comparable to the average levels observed in Sardo donkeys throughout the year. At Sardinian latitude, the month of June represents the culmination of the positive photoperiod with a maximum of daylight duration of nearly 15 hours/day. This was associated with the fact that, under comparable conditions of dietary β‐carotene from naturally available vegetation, Asinara donkeys display higher levels of circulating retinol than Sardo breed donkeys do, which can, however, rely on melanin for photoprotection.

**Figure 3:**
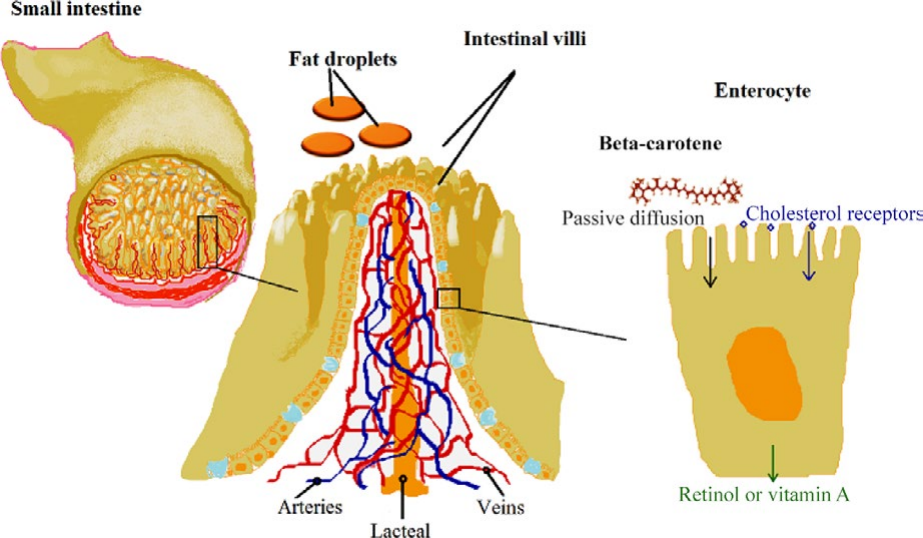
Scheme of β‐carotene fate as precursor to retinol (Vitamin A). The conversion is operated in the brush border of the enterocyte of the small intestine. Circulating β‐carotene around the limit of sensitivity suggests that donkeys are efficient converters of the provitamin into retinol. Blood serum concentrations of retinol turned out to differ in Asinara versus Sardo donkeys during positive photoperiod, as an adaptive metabolic measure to overcome the lack of melanin in specimens of albino breed. Retinol levels are suggestive of a pulsed mobilization of retinol into the bloodstream from liver stores in Asinara white donkeys

Dietary β‐carotene is consumed normally with fat compounds of the diet. Thus, β‐carotene follows dietary fat digestion and absorption processes. In the herbivore, the pro‐vitaminic β‐carotene is absorbed with vegetal fats in the small intestine. In particular, the absorption of β‐carotene from mixed micelles in the chymus of the small intestine occurs in the brush border of the enterocyte (Figure 3). β‐Carotene absorption can follow two fashions, but the detailed mechanisms ruling on which pathway may be preferable is not elucidated to date. Indeed, the literature reports (Amengual et al., 2011; Ford, Clinton, von Lintig, Wyss, & Erdmann, 2010) that BCOD2 rapidly metabolizes nonproteinoid carotenoids. However, it is well known that β‐carotene is not fully converted into retinol and that the conversion is self‐modulated according to the level of retinol already synthesized (Haskell, 2012; Tang, 2012). One mechanism of absorption is represented by the passive diffusion of β‐carotene through the mucosal layer of the intestine into the *vasum chyliferum,* which conveys fatty nutrients in the lymphatic circulation. Alternatively, the absorption can occur through cholesterol receptors expressed on the cell membrane of the brush border of the enterocyte (Haskell, 2012). In addition, interactions with other nutrient levels may impair β‐carotene absorption. The literature reports that nutritional deficiencies of iron, zinc, and protein may also affect estimates of the vitamin A equivalency of β‐carotene (Haskell, 2012). As to elements, Iron deficiency disrupts retinol homeostasis and results in decreased mobilization of vitamin A from the liver and low serum retinol concentrations in rats (Jang, Green, Beard, & Green, 2000). Marginal zinc deficiency results in a significant reduction in β‐carotene absorption in rats (Noh & Koo, 2003) and may also limit production of retinol‐binding protein and interfere with retinol homeostasis. Indeed, protein deficiency too is associated with reduced intestinal conversion of β‐carotene to vitamin A in rats (Gibson, 2007) and may interfere with production of chylomicrons, lipoproteins, and retinol‐binding proteins, with potential impacts on retinol metabolism (Gibson, 2007; Haskell, 2012; Jang et al., 2000; Noh & Koo, 2003). In both breeds, no nutrients were found to be below the minimum level of the physiologic range. Moreover, in Asinara white donkeys, no statistically significant correlations with retinol levels and zinc or total protein concentrations in blood serum were found. The comparison between the overall conditions observed in both groups of animals allowed us to draw several conclusions about the nutritional assessment, supported by nutrition‐related metabolic profiles. As a matter of fact, nutritional deficiencies with a direct impact on coat and skin health may involve polyunsaturated fatty acids and fat soluble vitamins (Jones et al., 1994; Vahlquist et al., 1982). The high concentration of retinol in the bloodstream during positive photoperiod can be due to the increased mobilization of retinyl esters stored in the liver. However, as neither intestinal nor hepatic mechanisms of retinol biosynthesis or mobilization were investigated in Asinara white donkeys, it is assumed that *in vivo* circulating retinol levels reflect the need to restore hematological levels in case of augmented need of photoprotection. The photoperiodism can strongly influence some important physiological functions of animals, in particular of wild and feral animals. For example, the mechanism behind the stimulus in relation to photoperiod and the retinal stimulation by daylight is capable to modulate the neuroendocrine retinal‐pineal‐gonadal axis. That way, cyclic reproduction of many animal species is influenced under seasonal control. In a similar way, though with different goals, retinol levels in the bloodstream of albino donkeys of Asinara breed can be modulated by photoperiodism, via stimulation of a more susceptible retina to natural light intensity and duration, in order to guarantee photoprotection of exposed tissues.

## 5 Conclusions

Higher blood serum concentrations of retinol in Asinara donkeys (albino breed) were found in relation to positive photoperiod, than found in blood serum from donkeys of Sardo breed (grey coated) involved in this trial. The concentration of this nutrient‐related metabolite can represent an alternative way to the lack of melanin in tissues, to explain the adaptation of albino donkeys in the natural Mediterranean environment. In this case, retinol may be an adaptive metabolic key to overcome the higher susceptibility to sun radiation of albino animals. Surprisingly, this peculiar form of albinism extended to all individuals of Asinara breed appears adapted to the environment. Probably, photoprotection might be achieved through higher levels of available retinol in the bloodstream capable to reach peripheral tissues. *In vivo* determination of high serum concentration of retinol in the Asinara donkeys paves the way to further investigations on the specific pathways leading to unpigmented skin protection from exposure to sun radiation.

## Conflict of interest

Authors declare no conflict of interest exists.
